# Changes in Site of Death Among Older Adults Without a COVID-19 Diagnosis During the COVID-19 Pandemic

**DOI:** 10.1007/s11606-023-08482-z

**Published:** 2023-11-09

**Authors:** Hiroshi Gotanda, Jessica J. Zhang, Debra Saliba, Haiyong Xu, Yusuke Tsugawa

**Affiliations:** 1 Division of General Internal Medicine, Cedars-Sinai Medical Center, Los Angeles, CA USA; 2 Department of Medicine, David Geffen School of Medicine at UCLA, Los Angeles, CA USA; 3 Geriatric Research, Education and Clinical Center, VA Greater Los Angeles Healthcare System, Los Angeles, CA USA; 4 Borun Center for Gerontological Research, UCLA, Los Angeles, CA USA; 5 RAND Health, Santa Monica, CA USA; 6 Division of General Internal Medicine and Health Services Research, David Geffen School of Medicine at UCLA, Los Angeles, CA USA; 7 Department of Health Policy and Management, UCLA Fielding School of Public Health, Los Angeles, CA USA

**Keywords:** COVID-19 pandemic, site of death, end-of-life care

## Abstract

**Background:**

Understanding how the coronavirus disease 2019 (COVID-19) pandemic affected site of death—an important patient-centered outcome related to end-of-life care—would inform healthcare system resiliency in future public health emergencies.

**Objective:**

To evaluate the changes in site of death during the COVID-19 pandemic among older adults without a COVID-19 diagnosis.

**Design:**

Using a quasi-experimental difference-in-differences method, we estimated net changes in site of death during the pandemic period (March–December 2020) from the pre-pandemic period (January–February 2020), using data on the same months in prior years (2016–2019) as the control.

**Participants:**

A 20% sample of Medicare Fee-for-Service beneficiaries aged 66 years and older who died in 2016–2020. We excluded beneficiaries with a hospital diagnosis of COVID-19.

**Main Measures:**

We assessed each of the following sites of death separately: (1) home or community; (2) acute care hospital; and (3) nursing home.

**Key Results:**

We included 1,133,273 beneficiaries without a hospital diagnosis of COVID-19. We found that the proportion of Medicare beneficiaries who died at home or in the community setting increased (difference-in-differences [DID] estimate, + 3.1 percentage points [pp]; 95% CI, + 2.6 to + 3.6 pp; *P* < 0.001) and the proportion of beneficiaries who died (without COVID-19 diagnosis) in an acute care hospital decreased (− 0.8 pp; 95% CI, − 1.2 to − 0.4 pp; *P* < 0.001) during the pandemic. We found no evidence that the proportion of deaths in nursing homes changed during the pandemic.

**Conclusions:**

Using national data on older adults without a COVID-19 diagnosis, we found that site of death shifted toward home or community settings during the COVID-19 pandemic. Our findings may inform clinicians and policymakers in supporting end-of-life care during future public health emergencies.

**Supplementary Information:**

The online version contains supplementary material available at 10.1007/s11606-023-08482-z.

## INTRODUCTION

One of the most important patient-centered outcomes related to end-of-life care is dying at the preferred place,^1^ with many people preferring to be at home at the end of life.^2^ As patient-centered goal-concordant care has gained increasing attention, site of death has been extensively used as an important outcome measure.^3–5^ Multiple interventions have been implemented to improve end-of-life care, including the promotion of advance care planning and increased access to hospice and palliative care services, and previous literature has shown that the prevalence of death at home or in the community has increased gradually over time.^3–5^ However, it remains largely unknown whether the site of death changed during the coronavirus disease 2019 (COVID-19) pandemic.

Disruptions in health care during the COVID-19 pandemic might be associated with substantial shifts in where people died for conditions not related to COVID-19. Hospitals were inundated with critically ill COVID-19 patients, and people had limited access to medical care.^6–9^ Patients deferred medical care to avoid contracting COVID-19 in a healthcare setting.^6–8^ While the majority of the increased deaths during the pandemic were attributable to COVID-19, there were sizable increases in deaths from other leading causes of death, such as heart disease, unintentional injuries, and Alzheimer’s disease.^10^ It is possible that some individuals could not receive medical care, particularly inpatient care, increasing deaths at home.^8, 11–13^ Understanding how site of death changed during the COVID-19 pandemic at the national level will inform resource allocation policies to maintain the quality of end-of-life care during future emergencies.

In this context, we sought to evaluate the changes in site of death during the COVID-19 pandemic among those without COVID-19 diagnosis. To account for the seasonality as well as the underlying historical changes in site of death, we used the quasi-experimental difference-in-differences (DID) design using the data on the same months in prior years (2016–2019) as the control (and compared before [January–February] and during [March–December] the pandemic in 2020). We also examined whether the changes in site of death during the pandemic varied by beneficiaries’ medical conditions (cancer, chronic obstructive pulmonary disease [COPD], and dementia^3^) as well as race and ethnicity.

## METHODS

### Data Source and Study Participants

We used a 20% random sample of Medicare claims data (see Supplementary Figure S1 for the study participant flow chart). The Medicare claims data provide beneficiary characteristics including age, monthly Part A and B coverage status, indicators for chronic conditions based on the definitions by the Chronic Condition Data Warehouse,^14^ and validated death dates (available for over 99% of decedent beneficiaries^15^). We linked the Medicare claims data to the Minimum Data Set (MDS), a federally mandated clinical assessment of all residents in Medicare- or Medicaid-certified nursing homes, to identify deaths in nursing homes.^16^

We first identified beneficiaries 66 years and older who died in 2016–2020 with Medicare Fee-for-Service coverage during the last 6 months of life. We then excluded beneficiaries who died in the acute care hospital setting with a diagnosis of COVID-19 using the *International Classification of Diseases, Tenth Revision, Clinical Modification* (ICD-10-CM) diagnosis codes of B97.29 (from January 1 to March 31, 2020) or U07.1 (from April 1, 2020, to December 31, 2020) for the principal or secondary diagnosis in inpatient claims for the hospitalization during which they died.^17^ We excluded those who died before July 1, 2016, because we were not able to determine their Medicare Fee-for-Service coverage status during the last 6 months of life.

We also identified the following three subgroups of Medicare beneficiaries based on definitions by the Chronic Condition Data Warehouse: cancer (breast, colorectal, endometrial, lung, and prostate cancer), COPD, and dementia.

### Site of Death Variables

We assessed each of the following sites of death separately: (1) home or community (e.g., assisted living facility); (2) acute care hospitals (including critical access hospitals); and (3) nursing homes (including skilled-nursing facilities and long-term nursing homes). We used patient discharge status codes, revenue center codes, and provider numbers in Medicare claims data and MDS to determine the site of death (Supplementary Table S1). We also examined deaths in inpatient hospice care (defined as short-term management of acute pain or other symptoms provided in an inpatient setting, such as in an acute care hospital, a nursing home, or a hospice-owned inpatient facility), but the results of the DID analysis are not presented because of the violation of the parallel trends assumption.

### Adjustment Variables

We adjusted for beneficiary characteristics: age at the time of death (continuous), sex, race and ethnicity (non-Hispanic White, non-Hispanic Black, Hispanic, or Other), 27 comorbidities (Chronic Condition Data Warehouse conditions), median annual household income estimated from residential zip codes (categorized into quintiles), and Medicaid coverage (i.e., dual eligibility). We also adjusted for fixed effects for Hospital Service Areas (HSAs), a collection of ZIP codes whose residents receive most of their hospitalizations from the hospitals in that area,^18^ to account for the geographic variation of the site of death (effectively examining the effect of the COVID-19 pandemic on site of death within the same HSA).

### Statistical Analysis

First, we described the characteristics of our study sample and plotted the monthly trends of the proportions of each site of death by year of death. Second, we estimated changes in the proportions of site of death during the pandemic compared to the pre-pandemic period using the DID analysis (using the beneficiary-level data). Given that the stay-at-home orders became effective in March 2020 in most states in the USA,^19^ we defined two binary indicators: (1) *Post_month*, coded as 1 if a beneficiary died between March and December and 0 if died in January or February, and (2) *Treatment_year*, coded as 1 if a beneficiary died in 2020 and 0 if died in 2016–2019. We regressed each site of death outcome on *Post_month*, *Treatment_year*, and their interaction term *Post_month* × *Treatment_year*, adjusting for beneficiary characteristics and HSA fixed effects. The coefficient of the interaction term represents the DID estimate of how site of death changed during the COVID-19 pandemic. We used the linear probability model (fitting an ordinary least squares regression model to binary outcomes using the heteroscedasticity-robust standard errors) because it allows a better interpretation of the coefficients of the interaction term.^20–23^ Lastly, we tested the parallel trends assumption for the DID analysis by comparing the pre-existing trends during the pre-pandemic months (January and February) in 2020 vs. 2016–2019.

### Subgroup Analysis

We conducted two subgroup analyses. First, we conducted a subgroup analysis by patients’ medical condition (i.e., cancer, COPD, and dementia) because the changes during the COVID-19 pandemic might vary between conditions. Second, we conducted a subgroup analysis by patients’ race and ethnicity because racial and ethnic minoritized patients might be disproportionately affected by the COVID-19 pandemic, and their experiences during the pandemic might have been different from White patients.

### Secondary Analysis

We conducted three secondary analyses. First, to test whether our findings are sensitive to the model specification, we fit a multinominal logistic regression model using deaths at home or in the community setting as the reference group, instead of linear probability models. Second, we conducted a stratified analysis by skilled-nursing (post-acute) vs. long-term care status among beneficiaries who died in nursing homes because the impact of the pandemic might have differed between these two distinct patient populations (see Supplementary Table S1 for the definitions of skilled-nursing and long-term care). Lastly, we used the event study design, instead of the DID design, to characterize changes during the pandemic months (March–December). We fit regression models that include dummy variables for each month, dummy variables for each year (2016–2020), and interaction terms between *Treatment_year* (i.e., a binary indicator for the year 2020) and dummy variables for each month, additionally adjusting for beneficiary characteristics and HSA fixed effects. The coefficients of the interaction terms represent the event study estimates, which are the net changes in the proportion of each site of death for each month while controlling for fixed differences between the year 2020 vs. 2016–2019 and monthly trends.

All analyses were performed using Stata/MP 16.1. The University of California, Los Angeles Institutional Review Board approved this study.

## RESULTS

### Beneficiary Characteristics

Our study included 1,133,273 beneficiaries (mean [SD] years of age, 82.7 [9.0]; 53.8% female), of whom 18.3%, 29.1%, and 52.0% had a diagnosis of cancer, COPD, and dementia, respectively (Table 1). We excluded 13,438 beneficiaries who died in acute care hospitals with COVID-19 before arriving at the final sample size (Supplementary Figure S1). The proportion of beneficiaries who died in 2020 was 23.2%, which was larger than the proportions of those who died in 2017, 2018, or 2019.

**Table 1:** Medicare Decedent Characteristics by Year of Death

Characteristics	Entire sample (*n* = 1,133,273)	Decedents in 2020 (*n* = 257,338)	Decedents in 2016–2019 (*n* = 852,697)
Age, mean (SD), y	82.7 (9.0)	82.7 (9.0)	82.70 (9.0)
Female, no. (%)	609,418 (53.8)	139,718 (53.2)	469, 700 (54.0)
Race and ethnicity, no. (%)			
Non-Hispanic White	948,794 (83.7)	217,336 (82.8)	731,458 (84.0)
Non-Hispanic Black	91,777 (8.1)	21,714 (8.3)	70,063 (8.1)
Hispanic	51,490 (4.5)	12,883 (4.9)	38,607 (4.4)
Other	41, 212 (3.6)	10,689 (4.1)	30,523 (3.5)
Annual median household income, mean (SD), $	66,477 (27,255)	66,940 (27,255)	66,337 (27,255)
Medicaid coverage, no. (%)	239,774 (21.2)	58,228 (22.2)	181,546 (20.9)

Values are among a 20% random sample of Medicare Fee-for-Service beneficiaries who died in 2016–2020 (excluding those who died before July 1, 2016)

### Time Trend of Site of Death

**Figure 1:**
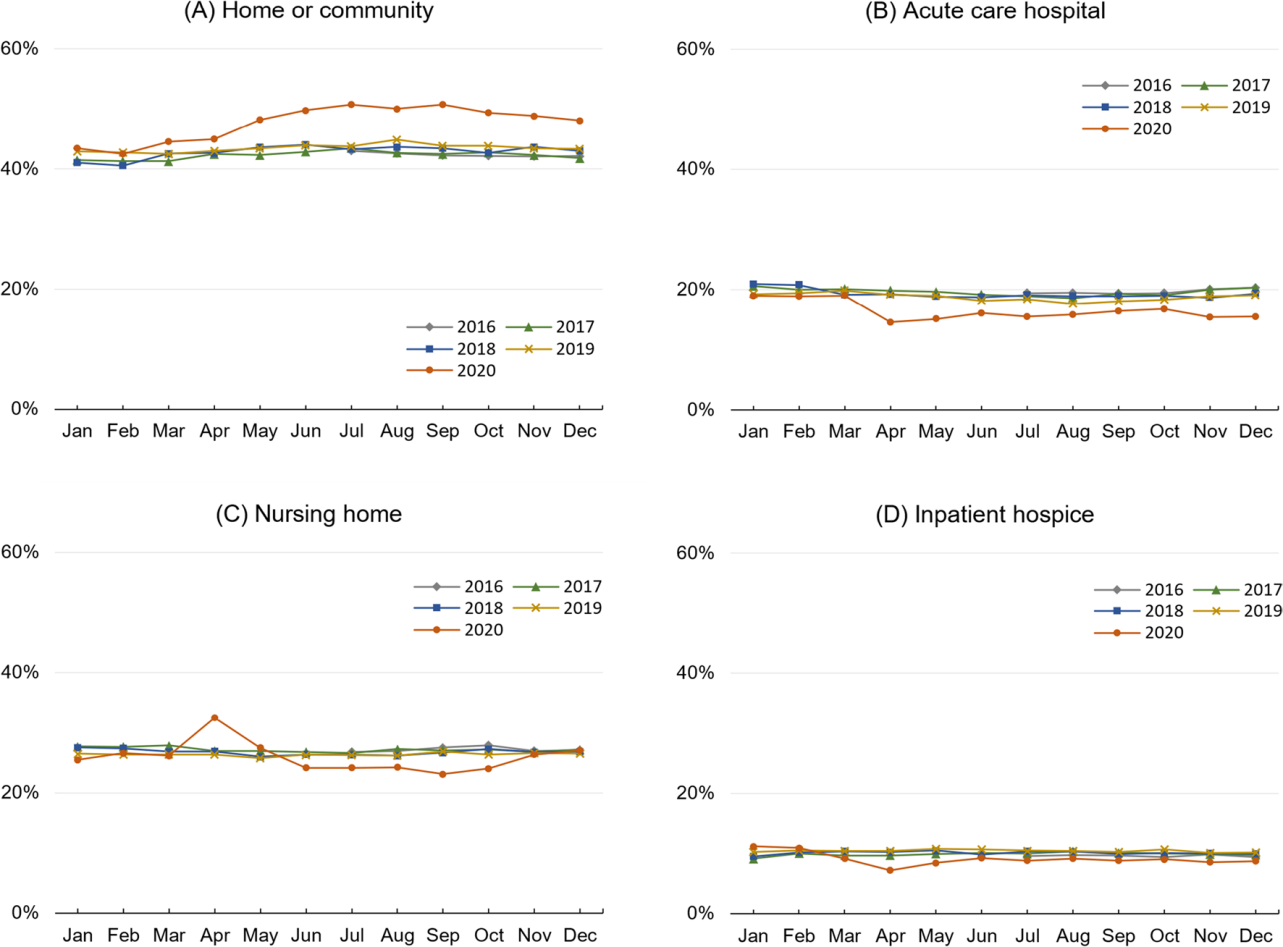
Time trend of site of death, 2016–2020. Notes: Data shown are unadjusted proportions of beneficiaries who died in each site of death by year of death based on a 20% random sample of Medicare Fee-for-Service beneficiaries who died in 2016–2020. We excluded beneficiaries who died in the acute care hospital setting with a diagnosis of COVID-19 and those who died before July 1, 2016 (see the main text for details)

The site of death was relatively consistent during years in the pre-pandemic period. However, the proportion of beneficiaries who died at home or in the community increased substantially when the pandemic started in March 2020 and continued throughout the rest of 2020 (Fig. 1). The proportion of those who died in acute care hospitals decreased in April 2020 and then stayed at a lower level throughout the rest of 2020.

### Changes in Site of Death During the COVID-19 Pandemic

Among beneficiaries who died without a hospital COVID-19 diagnosis, the proportion of those who died at home or in the community setting increased during the pandemic (DID estimate, + 3.1 percentage points [pp]; 95% CI, + 2.6 to + 4.0; *P*-value < 0.001) (Table 2) while the proportion of those who died in acute care hospitals decreased (DID estimate, − 0.9 pp; 95% CI, − 1.4 to − 0.5; *P*-value < 0.001). We found no evidence that the proportion of deaths that occurred among beneficiaries in nursing homes changed during the pandemic.

**Table 2:** Changes in the Proportions of Site of Death During the Pandemic Compared to the Pre-pandemic Period

	Difference-in-differences estimate, percentage points [95% CI]
Home or community	+ 3.1 [+ 2.6, + 3.6]
Acute care hospital	− 0.8 [− 1.2, − 0.4]
Nursing home	− 0.1 [− 0.5, + 0.3]

Presented values are based on a 20% random sample of Medicare Fee-for-Service beneficiaries who died in 2016–2020 (excluding beneficiaries who died in the acute care hospital setting with a diagnosis of COVID-19 and those who died before July 1, 2016). Difference-in-differences estimates are net changes in site of death during the pandemic period (March–December 2020) versus the pre-pandemic period (January–February 2020), using the data on the same months in the prior years (2016–2019) as the control. We used multivariable linear probability models adjusted for beneficiary characteristics including age at the time of death, sex, race and ethnicity, comorbidities, zip code level median annual household income, and Medicaid coverage, along with fixed effects for Hospital Service Areas. Given the violation of parallel trends assumption (see the main text and Supplementary Table S2), the results for inpatient hospice are not presented

Our test of the parallel trends assumption for the DID analysis showed no evidence that the pre-existing trends during the pre-pandemic months (January and February) differed in 2020 vs. 2016–2019 for the proportions of deaths at home or in the community setting, in acute care hospitals, and in nursing homes (Supplementary Table S2). However, the pre-existing trends differed for the proportion of deaths in inpatient hospice (i.e., violation of parallel trends assumption); therefore, the results of the DID analysis are not presented for this outcome.

### Subgroup Analysis

Time trends of site of death showed a similar pattern across three conditions except that there was an evident seasonality among beneficiaries with cancer and COPD (Supplementary Figure S2). We observed similar changes in site of death during the pandemic across three conditions, while the changes among those with cancer appear to be the largest (Table 3). The subgroup analysis by patients’ race and ethnicity showed that site of death shifted toward home or community during the pandemic among White beneficiaries but was less evident among Black beneficiaries, and the changes were not statistically significant among Hispanic beneficiaries (Table 4).

**Table 3:** Changes in the Proportions of Site of Death During the Pandemic Compared to the Pre-pandemic Period by Condition (Subgroup Analysis)

	DID estimate, percentage points [95% CI]		
	Cancer	COPD	Dementia
Home or community	+ 5.5 [+ 4.0, + 7.0]	+ 3.7 [+ 2.6, + 4.8]	+ 2.2 [+ 1.6, + 2.9]
Acute care hospital	− 0.3 [− 1.8, + 1.1]	− 0.8 [− 2.0, + 0.4]	− 0.6 [− 1.1, − 0.0]
Nursing home	− 1.2 [− 2.3, − 0.0]	+ 0.0 [− 0.9, + 0.9]	+ 0.3 [− 0.3, + 1.0]

Presented values are based on a 20% random sample of Medicare Fee-for-Service beneficiaries who died in 2016–2020 (excluding those who died before July 1, 2016). Difference-in-differences estimates are net changes in site of death during the pandemic period (March–December 2020) versus the pre-pandemic period (January–February 2020), using the data on the same months in the prior years (2016–2019) as the control. We used multivariable linear probability models adjusted for beneficiary characteristics including age at the time of death, sex, race and ethnicity, comorbidities, zip code level median annual household income, and Medicaid coverage, along with fixed effects for Hospital Service Areas. Abbreviations: *COPD*, chronic obstructive pulmonary disease; *DID*, difference-in-differences

**Table 4:** Changes in the Proportions of Site of Death During the Pandemic Compared to the Pre-pandemic Period by Race and Ethnicity (Subgroup Analysis)

	DID estimate, percentage points [95% CI]		
	White patients	Black patients	Hispanic patients
Home or community	+ 3.3 [+ 2.8, + 3.9]	+ 2.1 [+ 0.3, + 3.9]	+ 1.1 [− 1.3, + 3.5]
Acute care hospital	− 0.9 [− 1.3, − 0.4]	− 0.1 [− 1.7, + 1.6]	− 0.6 [− 2.8, + 1.6]
Nursing home	− 0.1 [− 0.6, + 0.3]	− 0.0 [− 1.5, + 1.4]	− 0.2 [− 1.9, + 1.6]

Presented values are based on a 20% random sample of Medicare Fee-for-Service beneficiaries who died in 2016–2020 (excluding beneficiaries who died in the acute care hospital setting with a diagnosis of COVID-19 and those who died before July 1, 2016). Difference-in-differences estimates are net changes in site of death during the pandemic period (March–December 2020) versus the pre-pandemic period (January–February 2020), using the data on the same months in the prior years (2016–2019) as the control. We used multivariable linear probability models adjusted for beneficiary characteristics including age at the time of death, sex, race and ethnicity, comorbidities, zip code level median annual household income, and Medicaid coverage, along with fixed effects for Hospital Service Areas. Abbreviations: *DID*, difference-in-differences

### Secondary Analysis

First, the sensitivity analysis using a multinominal logistic regression model showed similar results to the main analysis, except that the proportion of deaths in nursing homes decreased during the pandemic period, relative to deaths at home or in the community setting (Supplementary Table S3). Second, our stratified analysis by skilled-nursing vs. long-term care status among beneficiaries who died in nursing homes showed that their unadjusted trends both had a surge in April 2020 and a dip in the following months (Supplementary Figure S3), and there was no evidence that the proportions of deaths that occurred among these two groups changed during the pandemic (Supplementary Table S4). Lastly, the analysis using the event study design yielded similar results to the main analysis, except that there was an increase in the proportion of deaths in nursing homes in April 2020 followed by a decrease in August–October 2020 (Supplementary Figure S4).

## DISCUSSION

Using US nationally representative data on Medicare beneficiaries who died in 2016–2020 without a COVID-19 diagnosis, we found that site of death shifted from acute care hospitals toward home or community during the COVID-19 pandemic, with no changes in nursing home deaths. We observed similar changes in site of death during the pandemic among those with cancer, COPD, or dementia. The shift in site of death to home or community was observed among White beneficiaries but less evident among Black or Hispanic beneficiaries. These findings characterize how the COVID-19 pandemic affected site of death, and can inform clinicians and policymakers in responding to widespread disruptions in health care to support all people in dying in their preferred settings in the future.

Disruptions in health care during the pandemic are likely the primary driver of the observed shift toward deaths at home or in the community. While the number of deaths not attributable to COVID-19 increased during the pandemic,^10^ these deaths were more likely to occur at home or in the community during the pandemic compared to the pre-pandemic period. Surges in COVID-19 cases led to limited hospital resources, such as beds, staffing, and supplies, preventing patients from receiving appropriate medical care in hospitals that they would have received without the COVID-19 pandemic.^6–9^ In addition, patients feared being taken to hospitals and contracting COVID-19, leading to delays or reductions in presentation, for example, for acute myocardial infarction and stroke.^8, 11–13, 24, 25^ In future public health emergencies, public health authorities should emphasize the importance of seeking medical attention for severe symptoms and conditions to prevent unnecessary morbidity and mortality, along with support for caregivers, who had more involvement in patients’ health care and experienced more care burden during the pandemic.^26–28^ It is important to note, however, that our analysis may be overestimating the differences across settings because we could not exclude those who died with COVID-19 in non-acute hospital settings (e.g., at home or in the community) due to the unavailability of reliable data. Future studies using such data (e.g., death certificates) are warranted.

We did not find any change in the proportion of deaths in nursing homes. The main reason for this finding is likely that the surge in April 2020 and the dip in June–November 2020 (as observed in the unadjusted trend [Supplementary Figure S3]) canceled out in the DID estimates. In our DID analysis, we estimated the net changes in site of death during the pandemic period (March–December 2020) vs. pre-pandemic period (January–February 2020), using the data on the same months in the prior years (2016–2019) as the control. Therefore, this analysis is not able to evaluate changes during the pandemic months. Our sensitivity analysis using the event study design overcomes this limitation by providing the net changes for each month using the difference in February between 2020 vs. 2016–2019 as the reference. The event study design analysis showed that there was an increase in the proportion of deaths in nursing homes in April 2020 and a decrease in August–October 2020 (Supplementary Figure S4). These findings are probably explained by the disproportionate number of deaths that occurred in nursing home in April 2020^29^ and decreased utilization of nursing homes due to severe staffing and personal protective equipment shortages in the following months.^30, 31^ We also conducted stratified DID analysis by skilled-nursing (post-acute) vs. long-term care status among beneficiaries who died in nursing homes, and there was no evidence that the proportions of deaths that occurred among these two groups changed during the pandemic (Supplementary Table S4).

The shift in site of death during the pandemic toward home and community settings was consistent across medical conditions such as cancer, COPD, or dementia, while the changes among those with cancer appear to be the largest. There are two possible underlying mechanisms for this. First, limited access to inpatient hospice during the pandemic might have had a disproportionate impact on those with cancer who are more likely to use inpatient hospice compared to those with other chronic conditions.^32^ Second, those with cancer are less likely to be long-term nursing home residents than those with other chronic conditions,^33^ and, therefore, the proportion of death in long-term nursing homes may not have increased as much as seen in other chronic conditions (Supplementary Fig. 2).

In our stratified analysis by race and ethnicity, the shift in site of death toward home and community settings was less evident in Black than in White beneficiaries. The changes among Hispanic beneficiaries were not statistically significant. These may be due to disproportionately higher mortality from COVID-19 among racial and ethnic minorities.^34^

Our study adds to work from other countries that examined site of death during the COVID-19 pandemic. In the UK, O’Donnell et al. found an increase in deaths at home throughout the COVID-19 pandemic.^35^ Deaths where COVID-19 was reported on the death certificate contributed to an increase in care home and hospital deaths to a much larger extent than home deaths. Another study by Wu et al. using national registry data from England and Wales found that early in the pandemic there was an excess in mortality compared to expected deaths in care homes or hospice, of which 61% were related to COVID-19, and overall fewer deaths than expected in the hospital.^36^ However, these findings may not be applicable to the US population given that the degree of disruption in healthcare systems might have differed by country. We provide new evidence on site of death during the pandemic using a US nationally representative sample.

Our study has limitations. First, our study could not determine whether site of death was concordant with the patients’ goals. Some patients might have chosen to die at home or in the community and some of the increases in the proportion of death at home or in the hospital may not necessarily suggest healthcare disruption. Second, our findings may not be generalizable to site of death in later pandemic phases (i.e., after December 2020) due to changes in healthcare systems and consumer behaviors after vaccines began to be available. Future studies should examine whether the shift in site of death we observed persisted throughout the later phases of the pandemic. Lastly, our findings may not be generalizable to other populations such as younger populations or those who are covered by Medicare Advantage (managed care).

In summary, using a US nationally representative sample of Medicare decedents without a COVID-19 diagnosis, we found that site of death shifted toward home during the COVID-19 pandemic in 2020. Our findings characterize the effects of widespread healthcare disruption on site of death and may inform clinicians and policymakers in supporting end-of-life care during future public health emergencies.

### Supplementary Information

Below is the link to the electronic supplementary material.

Supplementary file1 (DOCX 1143 KB)
