# Supplementary material for: Changes in Site of Death Among Older Adults Without a COVID-19 Diagnosis During the COVID-19 Pandemic
Source: J Gen Intern Med. 2023 Nov 9;39(4):619–25. doi: 10.1007/s11606-023-08482-z (PMC10973311; doi:10.1007/s11606-023-08482-z)
Supplement: Supplementary file 1 — Supplementary file1 (DOCX 1143 KB) [file 11606_2023_8482_MOESM1_ESM.docx]

**Supplementary Appendix:**

**Changes in site of death among older adults without a COVID-19 diagnosis during the COVID-19 pandemic**

**Table of Contents**

[Supplementary Table S1. Definitions of site of death based on Medicare claims data and MDS 2](#_Toc147208512)

[Supplementary Table S2. Test of parallel trend assumption 3](#_Toc147208513)

[Supplementary Table S3. Difference-in-differences estimates using a multinominal logistic regression model 3](#_Toc147208514)

[Supplementary Table S4. Stratified analysis by skilled-nursing vs. long-term care status among beneficiaries those who died in nursing homes 3](#_Toc147208515)

[Supplementary Figure S1. Study participant flow chart 4](#_Toc147208516)

[Supplementary Figure S2. Unadjusted trends of the proportions of site of death 2016-2020 by condition 5](#_Toc147208517)

[Supplementary Figure S3. Unadjusted trend by skilled-nursing vs. long-term care status among beneficiaries those who died in nursing homes 8](#_Toc147208518)

[Supplementary Figure S4. Changes in the proportions of site of death during the pandemic compared to the pre-pandemic period using the event study design 9](#_Toc147208519)

# Supplementary Table S1. Definitions of site of death based on Medicare claims data and MDS

|  | **File Used** | **Patient Discharge Status Code** | **Additional Criteria** | **Description of Subgroup** |
| --- | --- | --- | --- | --- |
| **Home or community** | Hospice File | 40 = “Expired at home (hospice claims only)”  42 = “Expired (place unknown) (Hospice claims only)” | - | Died at home or in the community with hospice |
|  | - | - | - | Does not belong to any other categories |
| **Acute care hospital** | Inpatient File | 20 = “Expired (patient did not recover).” | Provider Number:  0001-0879 = “Short-term (general and specialty)” or 1300-1399 = “Critical Access Hospitals (CAH)” | - |
| **Nursing home** (skilled nursing or post-acute care) | Hospice File | 41 = “Expired in a medical facility such as hospital, SNF, ICF, or freestanding hospice. (Hospice claims only)” | Revenue Center Code:  Other than 0656 = “Hospice services-general inpatient care (non-respite)” | Died in a SNF with hospice but did not require “general inpatient care” |
|  | SNF File | 20 = “Expired (patient did not recover).” | - | Died in a SNF without hospice |
| **Nursing home** (long-term care) | MDS | 08 (MDS 3.0 code) = “Deceased” | - | Died in a long-term care nursing home with or without hospice |
| **Inpatient hospice** | Hospice File | 41 = “Expired in a medical facility such as hospital, SNF, ICF, or freestanding hospice. (Hospice claims only)” | Revenue Center Code:  0656 = “Hospice services-general inpatient care (non-respite)” | - |
| **Other** | Inpatient File | 20 = “Expired (patient did not recover).” | Provider Number:  Other than 0001-0879 or 1300-1399 | Died in non-acute care hospitals (e.g., inpatient rehabilitation, psychiatric hospitals) |

**Abbreviations**: ICF, intermediate care facility; MDS, Minimum Data Set; SNF, skilled-nursing facility;

# Supplementary Table S2. Test of parallel trend assumption

|  | Difference in pre-existing trends,  percentage points per month [95% CI] |
| --- | --- |
| **Home or community** | -0.3 [-1.2, +0.7] |
| **Acute care hospital** | -0.3 [-1.0, +0.5] |
| **Nursing home** | -0.3 [-1.0, +0.5] |
| **Inpatient hospice** | +1.0 [+0.3, +1.6] |

**Notes**: We tested the parallel trend assumption for the difference-in-differences analysis by comparing the pre-existing trends during the pre-pandemic months (January and February) in 2020 vs. 2016-2019 using a similar regression model to the main analysis.

# Supplementary Table S3. Difference-in-differences estimates using a multinominal logistic regression model

|  | Difference-in-differences estimates (ratio of relative risk ratio) |
| --- | --- |
| **Home or community** | reference |
| **Acute care hospital** | 0.86 [0.83, 0.89] |
| **Nursing home** | 0.89 [0.86, 0.93] |
| **Inpatient hospice** | 0.75 [0.71, 0.78] |

**Notes**: We fit a multinominal logistic regression model using deaths at home or in the community setting as the reference group, instead of linear probability models.

# Supplementary Table S4. Stratified analysis by skilled-nursing vs. long-term care status among beneficiaries those who died in nursing homes

|  | Difference-in-differences estimate, percentage points [95% CI] |
| --- | --- |
| **Nursing home** | -0.1 [-0.5, +0.3] |
| Skilled-nursing care | -0.3 [-0.8, +0.1] |
| Long-term care | +0.3 [-0.0, +0.6] |

**Notes:** We conducted a stratified analysis by skilled-nursing (post-acute) vs. long-term care status among beneficiaries who died in nursing homes. See Supplementary Table S1 for the definitions of skilled-nursing and long-term care.

# Supplementary Figure S1. Study participant flow chart


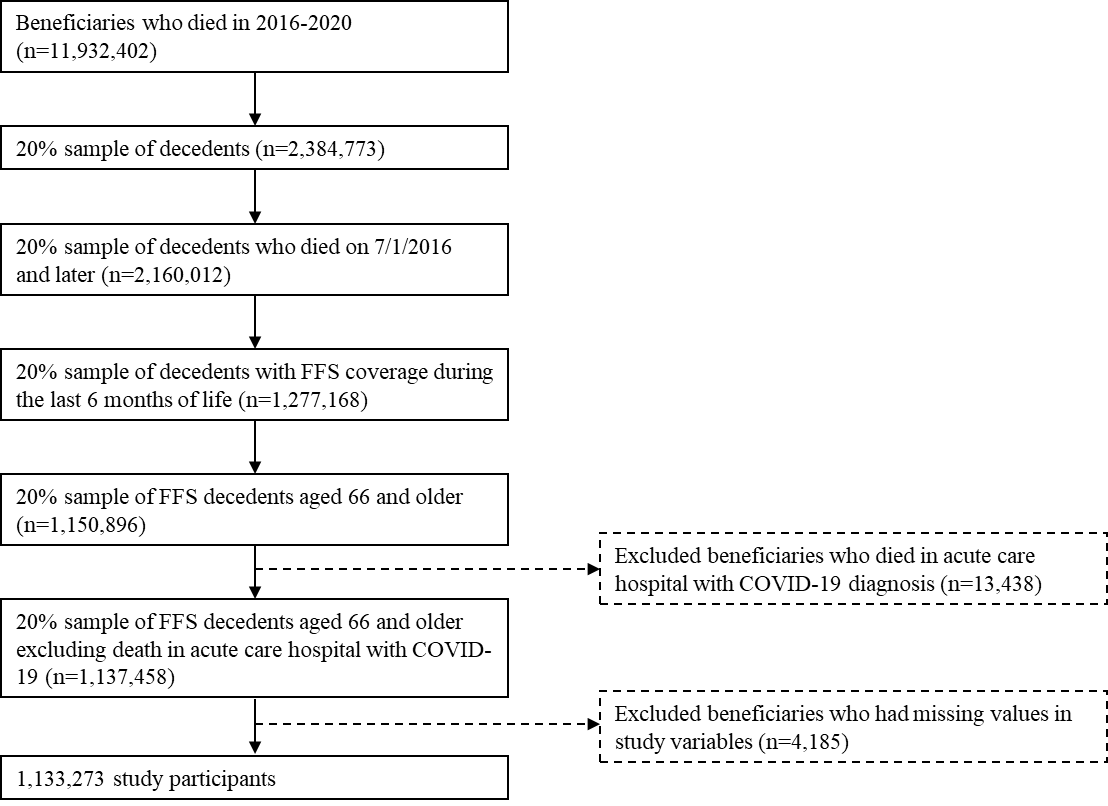


**Abbreviations**: FFS = fee-for-service.

# Supplementary Figure S2. Unadjusted trends of the proportions of site of death 2016-2020 by condition

**(1) Beneficiaries with cancer**


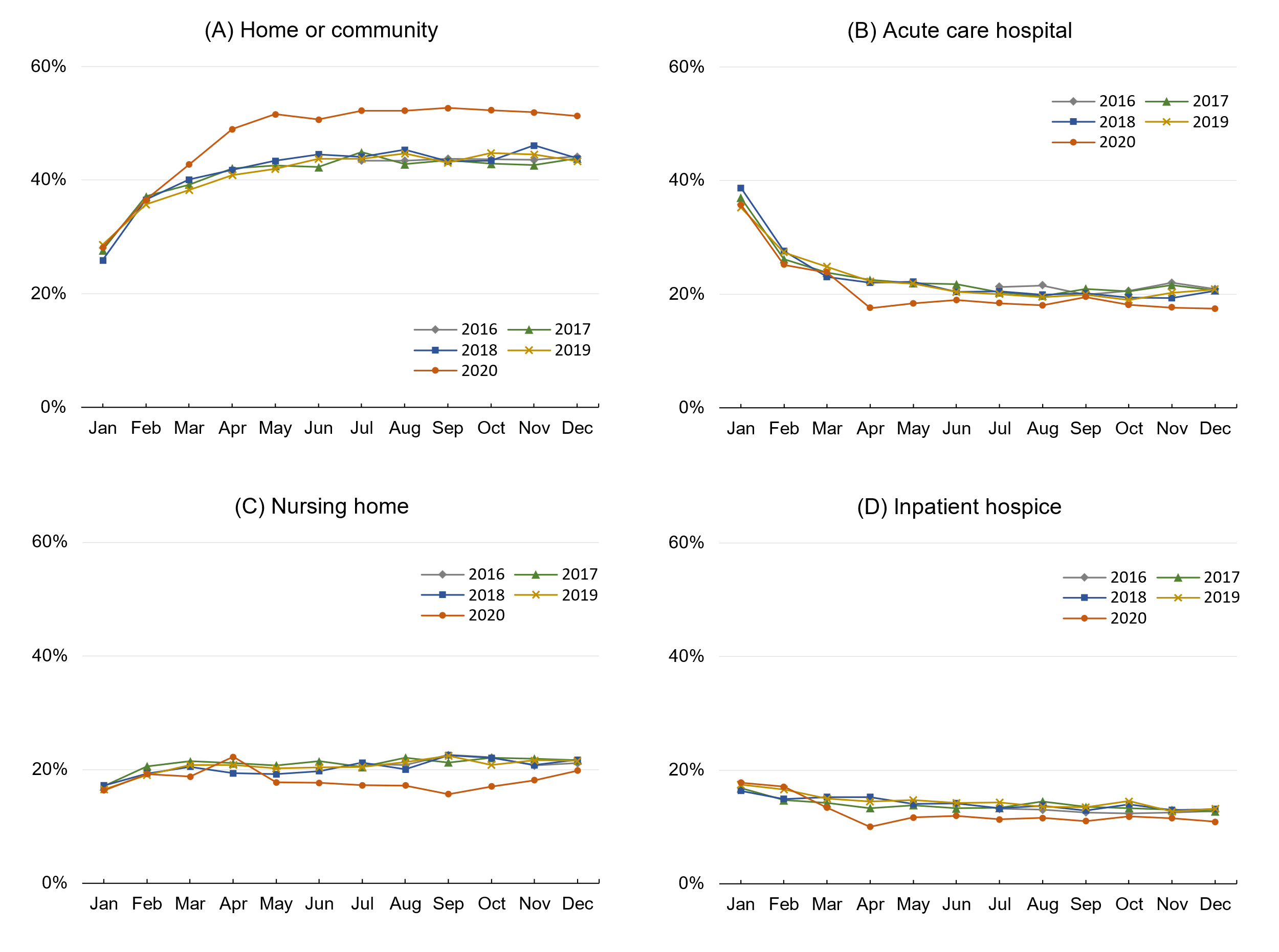


**(2) Beneficiaries with COPD**


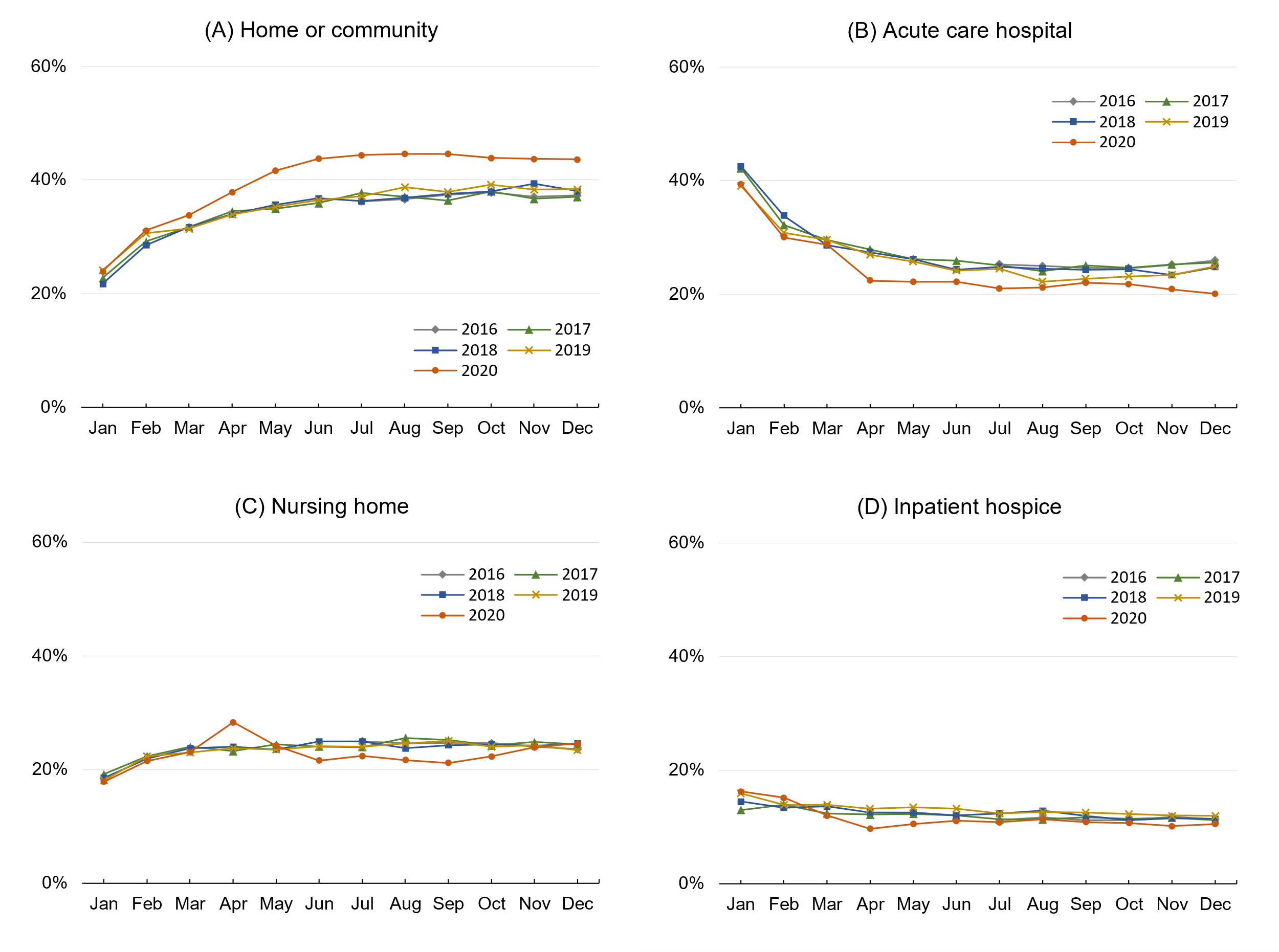


**(3) Beneficiaries with dementia**


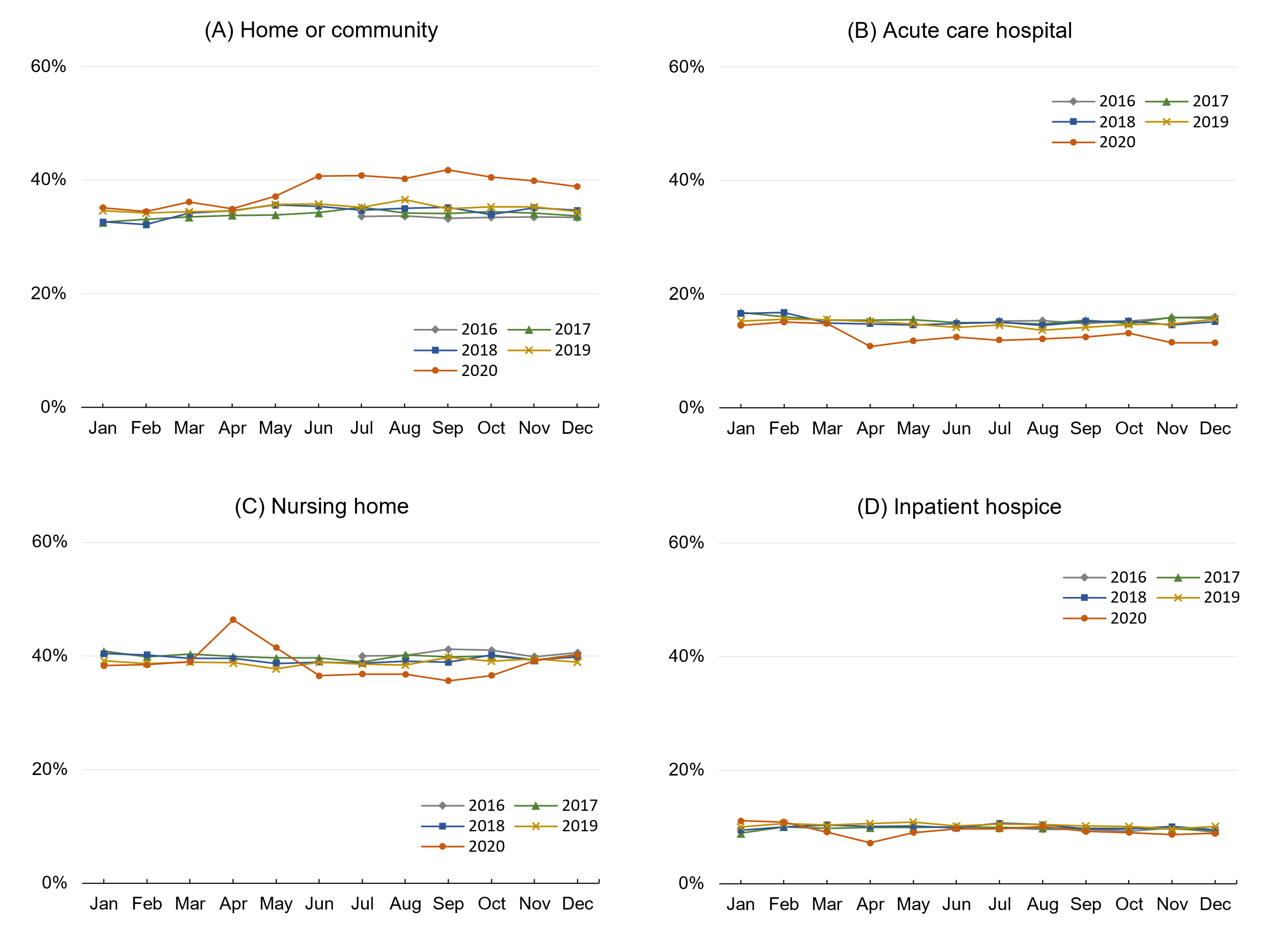


**Notes**: Data shown are unadjusted proportions of beneficiaries who died in each site of death by year of death based on a 20% random sample of Medicare fee-for-service beneficiaries who died in 2016-2020 (excluding those who died before July 1, 2016) for beneficiaries with (1) cancer, (2) chronic obstructive pulmonary disease (COPD), and (3) dementia.

# Supplementary Figure S3. Unadjusted trend by skilled-nursing vs. long-term care status among beneficiaries those who died in nursing homes

Deaths in nursing homes
 (long-term care)

Deaths in nursing homes
 (Skilled-nursing care)


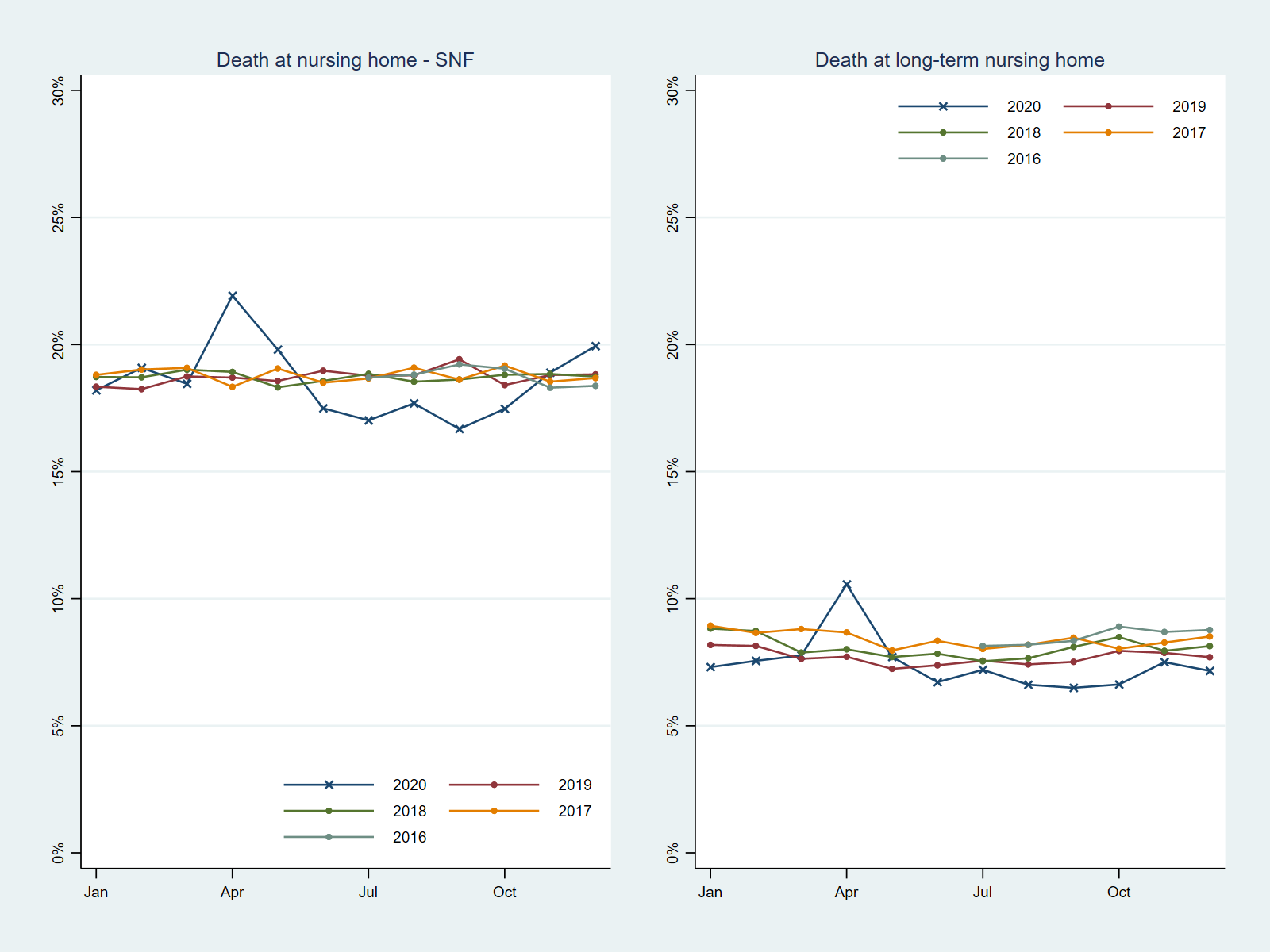


#
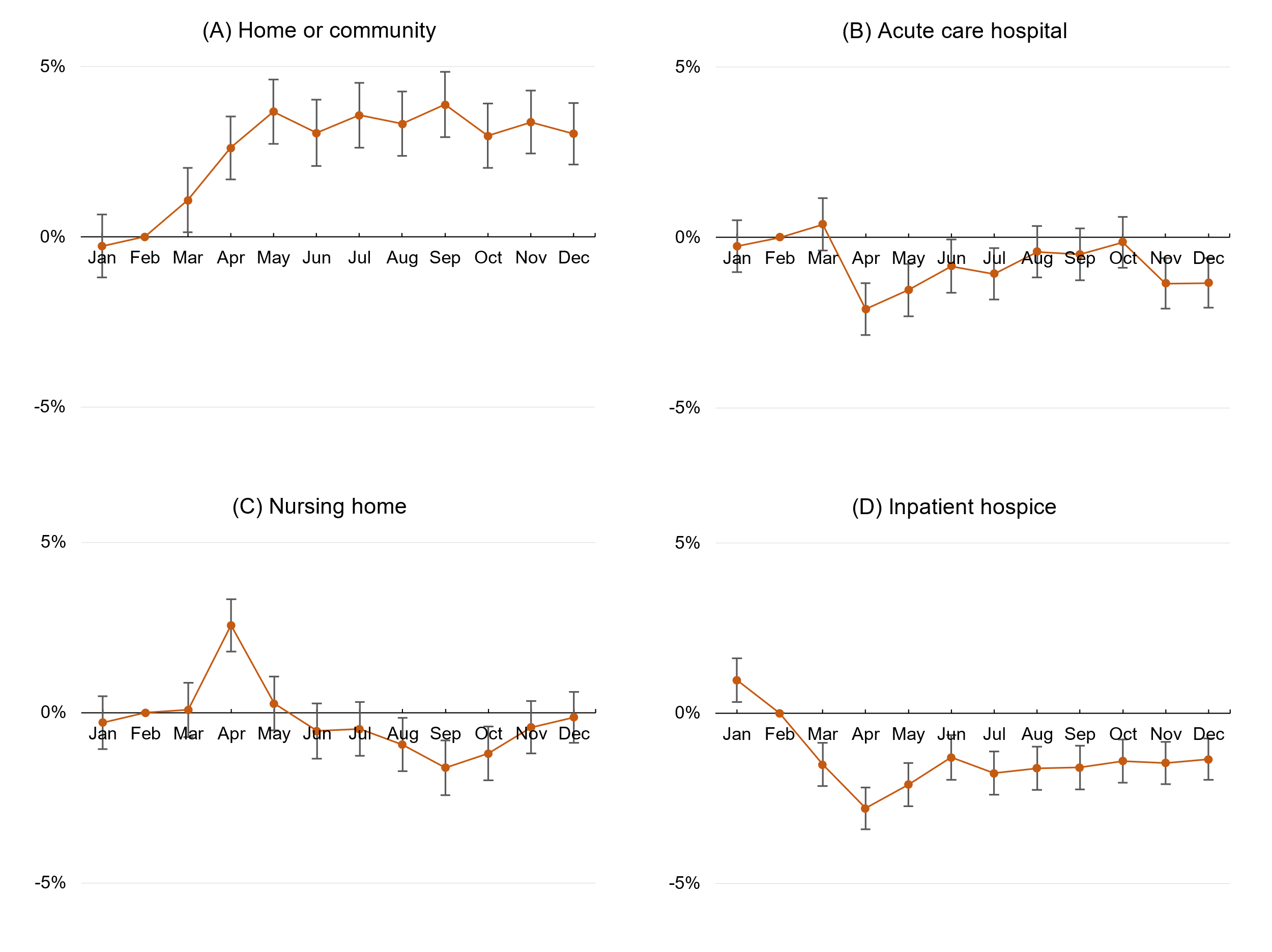
Supplementary Figure S4. Changes in the proportions of site of death during the pandemic compared to the pre-pandemic period using the event study design

**Notes:** We used the event study design, instead of the difference-in-differences design, to test whether our findings are sensitive to the model specification. See the main text for details.
